# Tuberculosis as Discussed at Portsmouth

**Published:** 1899-08-26

**Authors:** 


### Tuberculosis as Discussed at Portsmouth.

Among the many discussions which took place at Portsmouth during the meeting of the British Medical Association none were of wider interest than that on tuberculosis, and this not merely because of the importance of the subject itself, but from the fact that it was approached from many points of view, and was discussed in several of the sections almost simultaneously. It would be an exaggeration to suggest that anything really new was elicited, which, indeed, was hardly to be expected; but, take it altogether, a good general presentment was offered of the various views now current on the subject. Going to the root of the matter, it was generally accepted that all forms of tuberculosis are the outcome of an infection by the tubercle bacillus. To many it may seem that at this time of day it is hardly worth while stating so elementary a proposition. But the British Medical Association is a many-sided body, and it so happened that, while in the section of medicine the bacillus was received with all due respect, the official president of the section of public medicine threw scorn upon the microbe and all its ways. Hence a charming sense of variety, and much food for thought among the doubters. Dr. George Wilson, whose views, we presume, were pretty well known beforehand, defined his position in the following words: "The few pathogenic microbes which bacteriologists have discovered associated with human diseases . . . . are found associated with necrosed tissues, and it is open to argument whether, instead of being labelled the unconditioned *causæ* of these respective diseases, they may not be performing a benign function in changing the necrosed tissues into harmless products." He further went on to state that, "so far as preventive medicine is concerned, bacteriology has rather led us on false lines in assuming that the pathogenic microbe of any disease is the *causa causans* of that disease. I venture to say that the unconditioned microbe need have no terrors for humanity."

Coming to the practical application of his ideas in regard to tuberculosis, he said, "This insane hunt after the tubercle bacillus, as if it could be bottled up in twopenny-halfpenny spittoons and got rid of, is the insanest crusade ever instituted on illogical lines." How little it is true, however, that modern medicine accepts "the unconditioned microbe," the microbe minus its surroundings, as the cause of disease may be gathered from the address given by Professor Clifford Allbutt. Taking up the question, "Is phthisis catching?" Professor Allbutt said that undoubtedly it was catching in a sense, "but only in a limited sense." Indeed, it might be asked with much force how it was that nurses and others attending on patients suffering from phthisis did not catch the disease. The fact was that, ordinarily, it could not be taken, for even if introduced the bacillus would not live unless the soil were suitable. It was quite certain, he said, that the causation of the disease could not be summed up in the bacillus, valuable as its discovery had been; it was not even the immediate cause of the disease. Tissue proclivity and the proclivity of the individual played a very important part, and it was doubtful if it was ever absent in the etiology of phthisis. There was a remarkable difference in the proclivity of different animals to tuberculosis, and man would seem to have a strong resisting power, which was probably the reason why tubercle could not be considered contagious in the ordinary meaning of the word. In other words it is not the "unconditioned microbe," but rather the proclivity of the individual and the surroundings of the microbe which are of importance. In regard to the influence of proclivity, Sir William Broadbent asked how otherwise were they to explain why one case went to the bad and another did not? "It could not," he said, "be the number or quality of the bacilli." Thus we come back to what was so well described in the parable of the sower, nearly nineteen hundred years ago, for, in regard to microbes also, some fall "upon stony places" and take no root, while others fall "into good ground" and bring forth fruit an hundredfold. What, then, is "good ground"? What is "proclivity"? It is round this that just now the controversy centres. Sir Hugh Beevor aptly pointed out the constant association between phthisis and overcrowding. But do poverty, dirt, overcrowding, want of air and sunlight, and all the evil influences which we know to produce an excessive incidence of phthisis, do all these things act by producing a constitutional proclivity or merely by exposing the people who live amid such conditions to greater chances of infection by the microbe? If the latter, the prevention of phthisis is merely a matter of spittoons and cleanliness; if the former, far larger schemes of social reformation are required. And we think they are. We do not agree with Dr. Wilson, but we can hardly doubt that the extreme position which he took up will have served to emphasise what some people certainly of late have forgotten, namely, that, as Professor Allbutt put it, the causation of phthisis cannot be summed up in the bacillus.

